# Real-time phase-contrast x-ray imaging: a new technique for the study of animal form and function

**DOI:** 10.1186/1741-7007-5-6

**Published:** 2007-03-01

**Authors:** John J Socha, Mark W Westneat, Jon F Harrison, James S Waters, Wah-Keat Lee

**Affiliations:** 1 Advanced Photon Source, Argonne National Laboratory, 9700 S. Cass Ave., Argonne, IL 60439, USA; 2 Department of Zoology, Field Museum of Natural History, 1400 S. Lake Shore Dr., Chicago, IL, 60605, USA; 3 Section of Organismal, Integrative, and Systems Biology, School of Life Sciences, Arizona State University, PO Box 874501, Tempe, AZ, 85287-4501, USA

## Abstract

**Background:**

Despite advances in imaging techniques, real-time visualization of the structure and dynamics of tissues and organs inside small living animals has remained elusive. Recently, we have been using synchrotron x-rays to visualize the internal anatomy of millimeter-sized opaque, living animals. This technique takes advantage of partially-coherent x-rays and diffraction to enable clear visualization of internal soft tissue not viewable via conventional absorption radiography. However, because higher quality images require greater x-ray fluxes, there exists an inherent tradeoff between image quality and tissue damage.

**Results:**

We evaluated the tradeoff between image quality and harm to the animal by determining the impact of targeted synchrotron x-rays on insect physiology, behavior and survival. Using 25 keV x-rays at a flux density of 80 μW/mm^-2^, high quality video-rate images can be obtained without major detrimental effects on the insects for multiple minutes, a duration sufficient for many physiological studies. At this setting, insects do not heat up. Additionally, we demonstrate the range of uses of synchrotron phase-contrast imaging by showing high-resolution images of internal anatomy and observations of labeled food movement during ingestion and digestion.

**Conclusion:**

Synchrotron x-ray phase contrast imaging has the potential to revolutionize the study of physiology and internal biomechanics in small animals. This is the only generally applicable technique that has the necessary spatial and temporal resolutions, penetrating power, and sensitivity to soft tissue that is required to visualize the internal physiology of living animals on the scale from millimeters to microns.

## Background

The ability to visualize the internal anatomy of living animals is fundamental to our understanding of biology and medicine. Although imaging systems for respiratory, circulatory and musculoskeletal systems are available for large animals, real-time visualization of the internal processes of small animals has been limited by scaling factors and imaging technology. In order to visualize internal physiological mechanisms of millimeter-sized animals in real-time, a probe must have the following features: (1) ability to penetrate the opaque exterior, (2) spatial resolution in the 1–10 μm range, (2) temporal resolution below 100 ms, and (4) sensitivity to soft tissue. Visible light microscopy (conventional or confocal) is not broadly applicable for intact, live animals due to animal opacity and size limitations. Near-infrared (NIR) microscopy has been tried, but with limited success due to poor spatial resolution [1]. Magnetic resonance imaging (MRI) has been used to image insects [2], but the best resolution obtained so far is about 50 μm, and images must be averaged over seconds to minutes. For sufficient penetration, spatial resolution of ultrasound imaging is wavelength-limited [3] to about 100 μm. Conventional x-ray imaging relies on absorption as the contrast mechanism, which is ineffective at visualizing soft tissue. For example, at 25 keV, the maximum absorption contrast of a 100-μm diameter air-filled trachea in water is only 0.3%, smaller than the Poisson noise for a high-end 16-bit CCD camera (0.4%).

**Figure 1:**
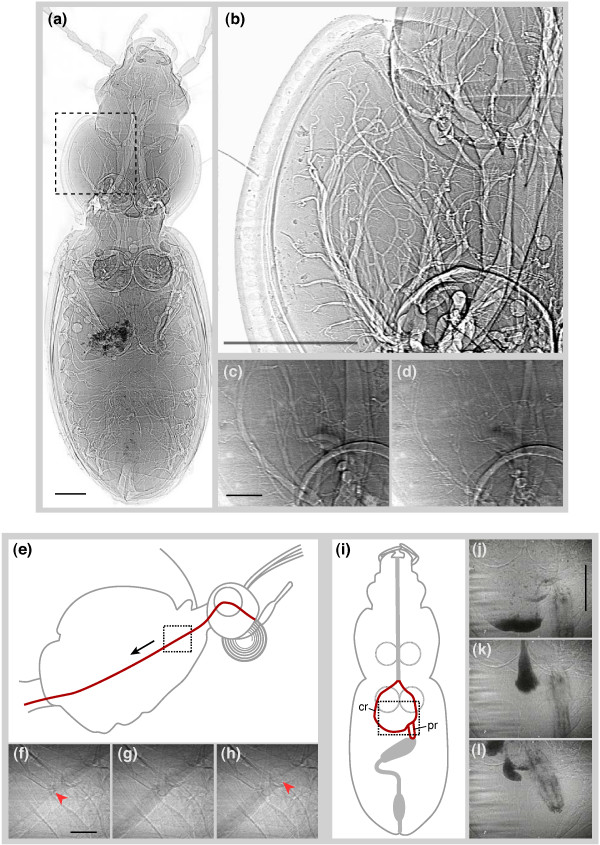
**Full-field 2-D projection images created using phase-contrast synchrotron x-rays. **Images were chosen to highlight the highest quality imagery currently obtainable (a, b) and corresponding stills from live video (c-l). (a) Carabid beetle (*Pterostichus stygicus*) in dorsoventral view with legs removed and sacrificed prior to imaging. Image is a high-resolution composite of multiple images. The air-filled tubes of the tracheal system can be prominently seen. The dark spots on the left side, mid-body are soil particles on the outer surface of the elytra. (b) Close-in view of one section of the prothorax, showing the branching pattern of tracheae. (c, d) One half-cycle of rhythmic tracheal collapse in a live carabid beetle (*Platynus decentis*) in dorsoventral view. Images are frame grabs from a video recording (See Additional file 1); time interval is 0.5 s. Total time of collapse and reinflation of the tubes is 1.0 s. (e-l) Visualization of internal food movement using labeled markers. (e) Schematic of the head and thorax of a butterfly (*Pieris rapae*) in lateral view. The foregut is shown in red; the square highlights the region of video stills in (f-h), and black arrow indicates the direction of food movement. (f-h) Video stills of passage of a food bolus posteriorly through the esophagus, moving through the frame from upper right to lower left (see Additional file 2). Red arrows indicate the leading (f) and trailing (h) edges of the bolus. Interval between frames is 0.5 s. Food is sugar water/iodine mixture. X-ray energy (33.2 keV) was tuned to just above the K-edge absorption band for iodine. (i) Schematic of a carabid beetle (*Pterostichus stygicus*) in dorsoventral view (legs removed). Circular structures in mid-body represent coxae; the gut is represented in gray and red. Square highlights video in (j-l), visualization of cadmium-laced food in the foregut (see additional file 3). Video stills (j-l) show movement of gut including anterior-posterior translation and squeezing of the crop (cr) and translation and rotation of the proventriculus (pr). The proventriculus is a valve that leads to the midgut [41]; here, it is closed. Note that only parts of the gut with contrast agent can be seen. Interval between frames: j-k, 2 s; k-l, 6 s. X-ray energy, 25 keV. Scale bars: a,b, 1 mm; c,d, 200 μm; f-h, 200 μm; j-l, 1 mm.

Compared to these other techniques, synchrotron x-ray phase-contrast imaging [4,5] is ideal for visualizing well-defined internal structures that have different mass densities. Tracheal tubes, in particular, show up extremely well (Figure 1a–d; see also Additional file 1), with edge contrast in a 100-μm diameter trachea that can be more than 50%. For example, this technique has been recently used to observe directly tracheal compression dynamics in opaque insects [6]. This research, which examined detailed networks of tracheal tubes down to tubes of 10 μm in diameter in living specimens, has revealed a mechanism of breathing that was previously identified with only a single species of translucent flea [7]. Such studies demonstrate the ability of synchrotron imaging to open up whole new avenues of scientific inquiry in biology.

**Figure 2:**
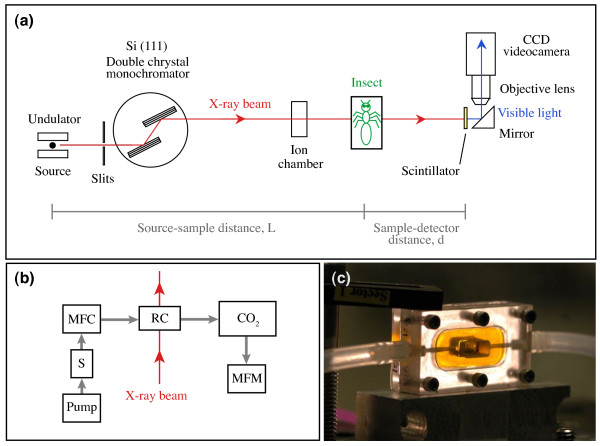
**Experimental setup. **(a) Schematic of phase-contrast imaging setup at the Advanced Photon Source. X-rays are produced by an undulator and monochromatized by a Si (111) double crystal monochromator. The partially coherent, monochromatic x-ray beam passes through an ion chamber and then the sample. The x-rays are converted to visible light by a scintillator screen, and the resulting image is recorded by a CCD image sensor. (b) Schematic of respirometry setup. MFC, mass flow valve and electronics control unit; S, CO_2 _scrubber; RC, respirometry chamber; CO_2_, CO_2 _analyzer; MFM, mass flow meter. (c) Typical plexiglass respirometry chamber. Yellow material is Kapton, used to provide an x-ray transparent window to the animal. Internal chamber volume is 0.25 ml.

**Figure 3:**
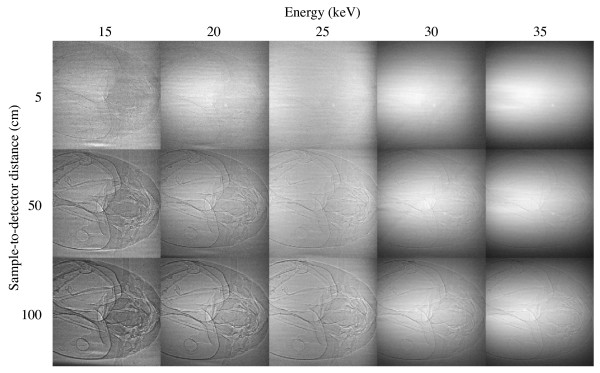
**Video image quality as a function of x-ray energy and sample-detector distance. **Data are from an ant head (*Camponotus pennsylvanicus*) using a Cohu 4950 video camera. Within each column, the absorbed x-ray dose on the insect is constant. For all images, the photon flux was kept at approximately 2 × 10^10 ^ph/s/mm^2^.

**Figure 4:**
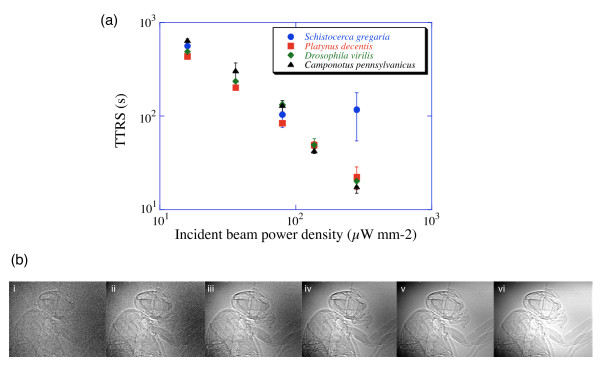
**Image quality versus TTRS. **(a) Plot of TTRS ('time to respiratory signal', which indicates major respiratory damage; see Figure 6) as a function of incident power density for all four species. At least three trials were performed per data point. A power law fit to the data gives: TTRS (s) = 90484 x^-1.02^, R = 0.97 where × is the incident beam power density in μW/mm^2^. TTRS measurements as a function of animal mass showed no correlation for the mass range 8.4–53.7 mg and 13.3–1473.5 mg for ants and grasshoppers, respectively. (b) Still images taken from video (16.6 ms exposure) footage of a dead fruit fly (*Drosophila melanogaster*) as a function of incident beam power density, which are, respectively from i-vi: 4, 8, 16, 36, 80, 103 μW/mm^2^. X-ray energy is 25 keV. At 80 μW/mm^2^, the photon density is 2 × 10^10 ^ph/s/mm^2^. Field of view is 1.0 × 1.3 mm using a 5× objective lens. Head and thoracic air sacs and leg trachea can be clearly seen. These images are taken with our new camera (Cohu 2700), which is twice as sensitive as the camera used in the major part of this study. Although we subjectively consider (iv) to be a high quality image, usable images can be obtained using lower beam intensities.

The basis of the x-ray phase-contrast imaging described here is Fresnel diffraction. For samples with minimal absorption, true for insects at the x-ray energies used here, the intensity of an image at a distance *d *downstream of the sample can be approximated by Equation 1 (see also [8]):

*I*(*x*, *y*) = *I*_inc _(1 + 1.3 × 10^-6 ^× *d *× *λ*^2 ^× ∇^2 ^[∫ *ρ*(*x*, *y*, *z*)*dz*]) * *R*(*x*, *y*)     (1)

where *I*_*inc *_is the incident beam intensity, *λ *(in Å) is the x-ray wavelength, *ρ *(in g cm^-3^) is the sample density, *R*(*x*, *y*) is the effective detector resolution, *x-y *is the image plane, *z *is the beam direction, and * denotes a convolution. *R*(*x*, *y*) depends on the detector properties (scintillator, lens and pixel size) and the projected source size, σsdL
 MathType@MTEF@5@5@+=feaafiart1ev1aaatCvAUfKttLearuWrP9MDH5MBPbIqV92AaeXatLxBI9gBaebbnrfifHhDYfgasaacH8akY=wiFfYdH8Gipec8Eeeu0xXdbba9frFj0=OqFfea0dXdd9vqai=hGuQ8kuc9pgc9s8qqaq=dirpe0xb9q8qiLsFr0=vr0=vr0dc8meaabaqaciaacaGaaeqabaqabeGadaaakeaadaWcaaqaaGGaciab=n8aZnaaBaaaleaacqWGZbWCaeqaaOGaemizaqgabaGaemitaWeaaaaa@329D@, where *σ*_*s *_is the source size, *d *is the sample-detector distance, and *L *is the source-sample distance (Figure 2). Increasing either the x-ray wavelength (lowering x-ray photon energy) or the sample-detector distance increases contrast (Figure 3). However, using longer x-ray wavelengths results in higher absorption, which is detrimental to the living animal. Similarly, increasing the sample-detector distance results in a loss of spatial resolution due to the increase in projected source size. Higher incident beam intensities give brighter and less noisy images (Figure 4b), but cause more harm to the insect. Given the complex interplay of these physical and biological factors, there is no *a priori *prescription for how best to optimize synchrotron phase-contrast imaging for organismal studies. Thus, one of the objectives of this study is to examine multiple experimental parameters to provide biologists a framework for using synchrotron phase-contrast imaging.

A major concern in using synchrotron x-rays to study physiological processes in small animals is the effect of the x-rays on the animal. Radiation causes molecular damage, including protein and lipid oxidation and gene transmutation; however, the effects depend on dose [9]. Previous studies show that fruit flies (*Drosophila melanogaster*) [10] and wasps (*Habrobracon and Bracon hebetor*) [11,12] temporarily lose motor control after a dose of about 1–2 kGy, but recover to normal behavior within minutes [9] or hours [12]. At exposures greater than 2.5 kGy, insects do not recover, although it is unclear when death actually occurs [12]. Feeding patterns are affected after 600 (*D. melanogaster*) [13] to 1000 Gy (*Bracon hebetor *Say) [11]. In one study of *D. melanogaster *receiving doses of 600 Gy, metabolic rates were unaffected one day *after *irradiation [13]. In summary, the literature suggests that there are no observable physiological effects at doses less than 500 Gy, a temporary loss of motor control is observed after ~1.5 kGy, and a more permanent loss of motor control occurs at doses greater than 2.5 kGy. However, in most prior studies of radiation effects on insects (concerned primarily with insect control [14] and ageing [15]), animals have been subjected to full body irradiation; the few studies that examined localized x-rays have used low levels of radiation [16-19]. Thus it is unknown how insects are affected by intense, targeted radiation – such as in a synchrotron x-ray beam – on specific parts of the body. Furthermore, previous studies focused primarily on effects that occur on a relatively long time scale, usually days after irradiation, and few studies have examined immediate radiation effects. This study strives to answer two questions: what combination of x-ray beam parameters optimizes image quality while minimizing damage to the animal? And under these conditions, how much time is available before the insect is negatively impacted? We varied x-ray parameters and used both CO_2 _emission patterns and motor behaviors as proxy indicators to assess physiological damage in four insect species. In addition, we demonstrate the range of studies that can be addressed using this technique by showing examples of high-resolution still imagery and real-time movement of food during ingestion and digestion.

## Results

There is a trade-off between image quality and survivorship: higher quality images require greater exposures to radiation, which result in greater harm to the animal. With our video camera and beamline configurations, we found a satisfactory comprise between image quality and survivorship by using 25 keV x-rays at 80 μW/mm^2 ^flux density (2 × 10^10 ^ph/s/mm^2^) and 1 m sample-detector distance (hereafter referred as 'nominal' settings). With these settings, insects exhibited no negative behavioral effects for a period of about 5 minutes. X-rays on the insect's head or thorax caused major changes to the respiratory pattern by about 17 minutes (2.4 kGy). With the beam on the abdomen, no significant changes were observed on the respiratory pattern throughout the full 2-hr trials (17.3 kGy), or even in two trials that were extended to 4 hrs (34.5 kGy). No thermal effects of the x-rays were observed. Food transport and gut structures could be clearly seen using labeled food (Figure 1e–l). In cases where tracking food transport was more important than maximizing the clarity of internal anatomy, 33.2 keV x-rays were successfully used to visualize iodine-laced food. Although not explicitly tested, the shorter wavelength of the x-rays at this setting results in lower absorption and therefore lower impact on the animals. We observed insect feeding under irradiation for more than 30 minutes, depending on species and location of the x-rays on the insect.

### Image quality

Figure 3 demonstrates the advantage of phase-contrast imaging over conventional absorption-based imaging. At *d *= 5 cm, where the phase effects are minimal, image contrast is poor for all energies, consistent with the fact that the absorption is small. For *d *= 5 cm and *E *= 15 keV, although some differences due to absorption can be seen, it is the small phase-contrast edge enhancements that make the features easily discernible. At a fixed energy, increasing *d *clearly increases the image contrast, as predicted by Equation 1. A careful comparison of the image at *d *= 100 cm with *d *= 5 or 50 cm shows that the spatial resolution of the *d *= 100 cm image is poorer: the line widths at the edges of the air sacs are broader in the *d *= 100 cm image.

### Thermal effects due to x-ray irradiation

Measurements from both the thermocouple and the infrared camera showed no change in temperature to the irradiated insect. This is not surprising, because the nominal power absorbed by the insect is, in the most extreme case, only about 20% of its unirradiated metabolic rate (Table 1).

**Table 1:** Measured no-beam metabolic rates and absorbed powers under x-ray irradiation (25 keV, 80 μW/mm^2^) for the four species studied.

**Species**	**Measured metabolic rate (no-beam, μW)**	**Measured absorbed power (μW)**
Grasshopper (adult)	2772 ± 248 (N = 2, 1250.0 ± 2.8 mg)	83.5 (head, N = 1, 1258.8 mg)
Grasshopper (juvenile)	230 ± 65 (N = 1, 64.3 mg)	26.0 (head, N = 1, 75.1 mg)
Beetle	441 ± 184 (N = 20, 46.6 ± 9.2 mg)	13.0 (head, N = 2, 40.1 mg)
Ant	127 ± 62 (N = 59, 21.4 ± 8.3 mg)	11.7 (head, N = 2, 19.2 ± 5.0 mg) 15.0 (thorax, N = 2, 19.2 ± 5.0 mg) 33.8 (abdomen, N = 2, 19.2 ± 5.0 mg)
Fruit fly	31 ± 9 (N = 28, 1.4 ± 0.3 mg)	1.3 (head, N = 2)

Except for grasshoppers, reported metabolic rate measurements are averages of all available no-beam CO_2 _recordings for each of the four species. For grasshoppers, metabolic rates were not averaged due to the wide mass distribution. Conversion from CO_2 _output to metabolic rate assumed an energy equivalence of 20.1, 24.5, 27.6 and 21.2 J/ml of CO_2 _for grasshoppers [37], beetles [38], ants [39], and fruit flies [40]. Except for grasshoppers and ant abdomen, the beam was larger than the part of the animal being irradiated. Under nominal beam intensities, the absorbed power due to the x-ray irradiation was less than 20% of the unirradiated metabolic rate. Values are mean ± SD.

### X-ray irradiation effects on CO_2_ emission patterns

**Figure 5:**
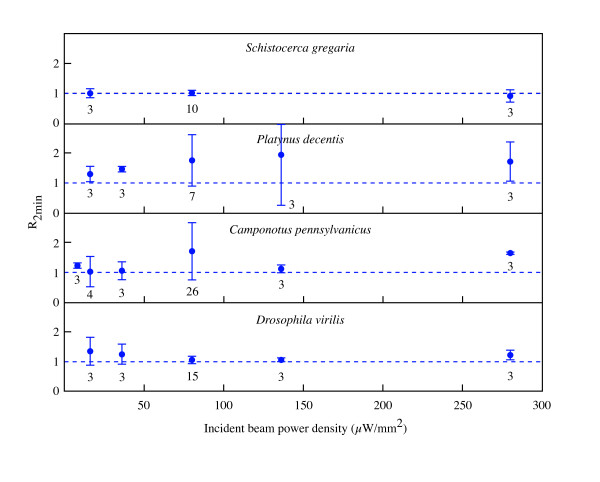
**R_2 min _as a function of incident beam power density. **R_2 min _is the ratio of mean CO_2 _emission rate during the 2 minutes *after *'beam on' divided by the mean CO_2 _emission rate during the 2 minutes *before *'beam on'. Error bars denote standard deviation; numbers below each data point correspond to sample sizes. The 25 keV x-ray beam was incident on the head.

The effect of x-ray radiation dosage on metabolic rates was quantified by examining the effect of incident beam flux density on the ratio of mean CO_2 _emission rate during the 2 minutes *after *'beam on' divided by the mean CO_2 _emission rate during the 2 minutes *before *'beam on' (*R*_2 *min*_) (all measurements at 25 keV; Figure 5). Except for grasshoppers, the data show a slight but significant increase in average CO_2 _emission immediately after 'beam on'. This small increase is probably the result of movement of the insect (seen in the x-ray videos) as it tried to move away from the beam. However, increasing beam intensity to four-times nominal values had little effect on *R*_2 *min*_, suggesting that, although insects appear to sense the beam, there is a considerable safety margin in the capacity to absorb x-rays before major physiological damage occurs during the initial minutes of exposure.

**Figure 6:**
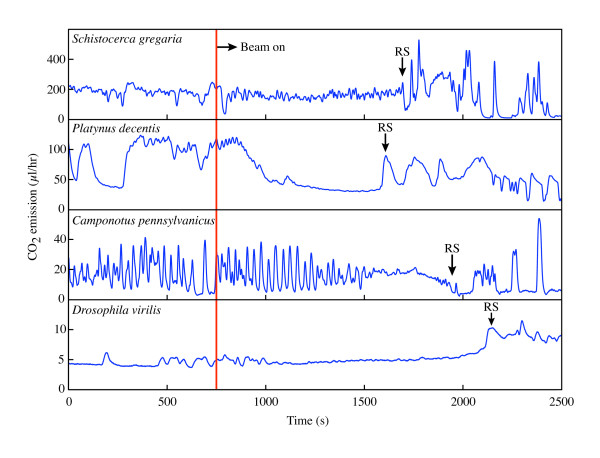
**Representative CO_2 _emission traces for the four species used in this study. **The x-ray beam (25 keV, 80 μW/mm^2^) was incident on the insect's head. No qualitative changes are seen immediately after beam on. A major change in CO_2 _emission pattern after 1000–1500 s of x-ray exposure is marked by RS (respiratory signature). The RS was based on CO_2 _emission patterns and was corroborated with x-ray video behavioral data; the RS is a major change in CO_2 _release pattern associated with shaking of the head or mouthparts. For the grasshoppers (*Schistocerca gregaria*), no behavioral change was observed at RS.

Although CO_2 _emission patterns during the first minutes after 'beam on' are similar to those prior to irradiation (Figure 6), a major change in the CO_2 _emission pattern (respiratory signal, RS) was observed in all species after 1000–1500 s of irradiation. The RS was correlated with dorsoventral head-shaking in the ants and beetles, and a quivering proboscis in fruit flies, but was not correlated with any observable behavior in grasshoppers. We note that for ants, the RS is qualitatively similar to the 'mortal fall' signature observed for ants under thermal stress [20], even though in our case there were no measurable temperature changes (0.1 K resolution) in the animal. Shortly after the RS, the CO_2 _emission pattern became periodic for all ant samples, and for most of the beetle (12 of 15) and grasshopper (9 of 16) samples. For ants, these periodic patterns resemble discontinuous gas exchange (DGC) reported in decapitated ants [21], so we interpret the RS as indicating major brain damage.

Time to respiratory signal (TTRS) varied strongly with incident beam power density (Figure 4a); higher power densities resulted in lower TTRS for all species. The one exception was grasshoppers at the highest power density, which showed a higher TTRS than the other species. Figure 4b shows still images taken from the video corresponding to the different incident power densities. Together, Figures 4a and 4b provide a guide for an experimenter to gauge the compromise between image quality and physiological impact.

### TTRS dependence on insect mass

Measurements at the nominal beam intensity showed no mass dependence on the TTRS for grasshoppers (N = 9, 13.3–1473.5 mg, Spearman ρ = 0.23, p = 0.51) and ants (N = 14, 8.4–53.7 mg, Spearman ρ = -0.24, p = 0.38). One possible explanation for this lack of pattern is that, in all cases, major portions of the brain were irradiated.

### TTRS dependence on x-ray beam location on the insect body

**Figure 7:**
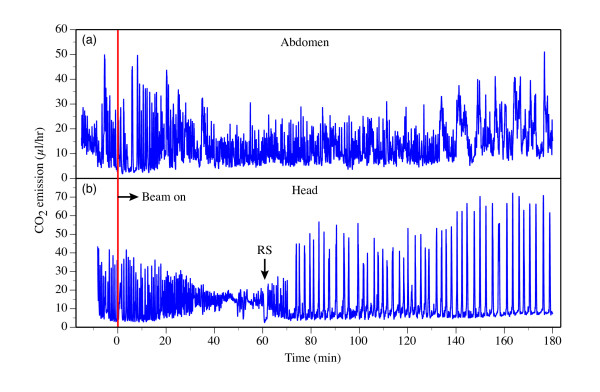
**Comparison of x-ray impact on two different regions of the insect body. **Representative CO_2 _traces are from two different ant specimens (*Camponotus pennsylvanicus*) with the x-ray beam targeted on the abdomen (a) and the head (b). Even though the abdomen-irradiated ant (a) received a higher x-ray flux (80 vs. 36 μW/mm^2^), it showed no discernible changes in CO_2 _emission pattern. In contrast, the head-irradiated ant (b) showed dramatic changes in CO_2 _emission, including a decrease in variance leading up to the RS (at which point the head stopped moving) and a cyclic pattern of release (similar to DGC in decapitated ants [25, 26]) thereafter.

From CO_2 _emission measurements in ants, there is no significant difference (Student's t-test, p = 0.4) in TTRS between having the nominal x-ray beam incident on the head (N = 3, TTRS = 1299 ± 177 s) or the thorax (N = 3, TTRS = 1092 ± 249 s) of the animal. In contrast, no change in CO_2 _emission pattern was observed with the x-ray beam incident on the abdomen (N = 3, Figure 7a), even after 4 hrs of exposure at the nominal beam intensity. These results are consistent with the fact that in ants, and most insects, most ventilatory activity is controlled by major ganglia of the central nervous system in the head and thorax [22].

### X-ray irradiation effects on motor function

Using simple behavioral assays, we tested for the presence/absence of righting behavior, defensive behavior, and locomotor ability after a fixed duration of x-ray exposure on the head using nominal beam settings (Table 1). No changes in behavior were observed within the first 5 minutes of exposure. During 6–25 minutes of x-ray exposure, ants, beetles and flies progressively lost motor abilities, starting with leg twitches and ranging to full immobility. By contrast, after 2 hrs of exposure, the grasshoppers could still right themselves, hop, feed and fly (and were later observed to mate and lay eggs). One major difference between the grasshoppers and all other insects studied is that, because of their large size, only a part of the grasshopper's head was irradiated as opposed to the entire head in the other taxa. We note that, consistent with other studies [11,12,23], the loss of locomotor abilities observed in the insects at lower dosages was temporary, indicating radiation-induced lethargy. In many individuals, we observed recovery minutes to hours later, suggesting that radiation damage was at least partially repairable.

## Discussion

Our measurements show that a major change in CO_2 _emission pattern, probably indicating major damage to the central nervous system, occurred after about 2.4 kGy when the insect was exposed on the head or thorax. No change in CO_2 _emission was observed if the x-ray beam was incident on the abdomen. The TTRS was independent of mass and species. In ants, beetles and juvenile grasshoppers whose entire heads were irradiated, a cyclic or discontinuous gas exchange (DGC) CO_2 _emission pattern [24] occurred after the RS. Ants have also been shown to exhibit DGC after they are physically decapitated [25,26], supporting the hypothesis that the x-ray treatment caused major brain damage. In cases where the RS was observed in this study, it is likely that the very high, acute dose of radiation caused profound tissue damage, causing such problems as potassium leakage [27,28] and leading to effects akin to the 'central nervous system syndrome' known from mammals [29]. One puzzling result is that although grasshoppers were no different in TTRS at some power densities, they showed a surprising degree of behavioral control after long periods of irradiation, suggesting a greater tolerance of x-rays to the head. For these animals, whose heads were larger than the size of the x-ray beam, the positioning of the x-ray beam may have missed or only partially damaged parts of the central nervous system, including the major ganglia controlling respiratory and motor function. In particular, partial control of motor behaviors such as walking occur in ganglia in the thorax [30-32]. Many of the smaller insects received incidental radiation on the thorax due to geometry during nominal 'head only' trials and exhibited motor loss, lending further weight to this hypothesis.

Due to the many factors that contribute to the question of image quality versus survivorship, there is no single set of x-ray parameters that provide an optimal setting. Generally, one would like a very small source size to minimize image blur, and an efficient detector system so that a less intense x-ray beam can be used to maximize survivorship. In practice, for insect physiology, the first question is whether the particular internal dynamic or morphology can be visualized by this technique. Given the particular source and detector that is available, one usually starts with parameters that give superior image quality. Based on our experience with insects, this is usually with an x-ray energy of 10–20 keV and a sample-detector distance of 10–100 cm. After the desired feature is visualized, the experimenter can optimize the system based on the relative importance of image contrast, spatial resolution, and survivorship.

With our commercially available standard NTSC interlaced video camera (30 fps, Cohu 4920) and nominal incident fluxes of 2 × 10^10 ^ph/s/mm^2 ^at 25 keV, a 16.6 ms (1/60 s) exposure time is sufficient to produce a quality image and record many physiological functions. For body functions that require shorter exposure times (e.g., flight), higher incident beam fluxes are necessary (and are available), in which case insect survivorship will be correspondingly reduced. However, in many cases the total time needed to record such rapid phenomena will be lower. Nonetheless, because the current overall detection efficiency is still very low (< 10%) [33], there is ample room for technological improvement with the development of better detectors. In fact, during the course of this manuscript preparation, we acquired a new video camera with the same pixel numbers and sizes, but with twice the sensitivity; thus, we can now obtain high-quality images with only 1 × 10^10 ^ph/s/mm^2 ^incident beam flux (Figure 4b). This improvement should *double *the working time (from 5 to 10 minutes) before any x-ray related effect is observed.

Finally, although this study was targeted specifically at insects, these species were chosen primarily as exemplars to introduce the technique to the biological community. Synchrotron x-ray phase contrast imaging is broadly applicable to any organism with features on the micron scale and above. However, we urge caution when exploring new systems with this technique; it is crucial to understand the effects of the radiation on the organism when making biological interpretations.

## Conclusion

Synchrotron x-ray phase contrast imaging shows great promise as a powerful new tool for internal visualization in biological and medical research. This is the only generally applicable technique that has the necessary spatial and temporal resolutions, penetrating power, and sensitivity to soft tissue that is required to visualize the internal physiology of small living animals on a scale from millimeters to microns. The impact of this technique is just beginning to be seen as it is applied to some of the more easily arranged experiments such as those on the respiratory systems of insects, where it has already had a major impact. The discovery of rhythmic tracheal compressive movements in taxa in which it was previously unknown [6] has opened whole new areas of research, for example those aimed at determining morphological mechanisms of compression and the role of associated convection in insect physiology and evolution. Another exciting possibility is the visualization of previously unknown, complex circulatory patterns within insects that have only been inferred before from changes in body surface temperature [34].

Current uses of the technique include the analysis of the rapidly moving internal mouthparts of biting insects and the visualization of fluid motion in the pumping organs of fluid feeding insects such as flies and butterflies. The ability to see inside the animal, including the internal workings of jaws, legs, and wing hinges, may be of significant utility in the exploration of functional diversity. Although more challenging due to lower density differences, this approach has also yielded impressive x-ray video of insect digestive (Figure 1e–l; see also Additional files 2 and 3) and circulatory system function, including the pumping of the tiny pulsatile organs that maintain the internal pressure of the antennae of ants. The first synchrotron research on living vertebrate musculoskeletal systems has recently begun with successful video of the interior bones of the pharynx and skull during fish respiratory pumping. The potential for investigation of model systems in genetics and medicine such as fly, zebrafish, and mouse is considerable, as the natural and normal mechanisms of heart, circulatory, digestive, and locomotor systems can be analyzed in new ways and compared to mutants or disease models that may be used to study human health concerns. Ultimately, the ability to clearly visualize internal functions in small animals will have a large impact in both biology and medicine.

## Methods

### Synchrotron x-ray phase-contrast imaging

Experiments were performed at the XOR-1ID and XOR-32ID undulator beamlines at the Advanced Photon Source (Figure 2). Synchrotron x-rays are produced here by a source with full-width half maximum dimensions of 35 μm (vertical) by 560 μm (horizontal) and source-to-sample distances of 60 m and 40 m, respectively. A Si (111) double crystal monochromator was used to select the x-ray wavelength. The incident beam flux (photons/s/mm^2^) was changed by varying the undulator magnetic gap and was monitored with an upstream ion chamber. Insects were mounted on top of a remotely controlled stage that enabled precise positioning in the x-ray beam. After passing through the insect, the x-rays were converted to visible light via a cerium doped yttrium aluminum garnet scintillator. The sample-to-scintillator distance was approximately 1 m; a distance of this magnitude is necessary for obtaining the phase-contrast effect. The visible light created by the scintillator was imaged onto a video camera (Cohu 4920 or Cohu 2700, Cohu, San Diego, CA, USA) or higher resolution CCD camera (SensiCam QE, Cooke, Romulus, MI, USA) using a 2× or 5× microscope objective. The field of view was 2.4 mm × 3.2 mm and 1.0 mm × 1.3 mm for the 2× and 5× objectives, respectively. Unlike most prior studies, the size of the x-ray beam was comparable to the size of the insect, and we only exposed parts of the insect to radiation.

### Animals

We conducted the majority of our experiments on carpenter ant workers (*Camponotus pennsylvanicus*, n = 59), but also explored taxonomic diversity by examining beetles (*Platynus decentis*, n = 20), fruit flies (*Drosophila virilis*, n = 28), and grasshoppers (*Schistocerca gregaria*, n = 19). Ants and fruit flies were purchased from Carolina Biological Supply Company (NC, USA). Beetles were collected in the woods at Argonne National Laboratory, and grasshoppers were reared at one of the author's laboratory (JH). Insects were housed with free access to food and water prior to experimentation.

### Survivorship and behavior

To determine the length of time that insects could withstand radiation on a particular part of the body (head, thorax, or abdomen), insects were monitored for CO_2 _release using flow-through respirometry while being observed with x-rays (Figure 2b). Insects were cold anaesthetized and placed individually in custom plexiglass respirometry chambers (volumes: 0.03, 0.25, 1.0, and 9.5 ml) with Kapton (Dupont, DE, USA) windows for x-ray transmission (Figure 2c). Because some insects actively moved away from the beam upon contact, cotton was used to fill in gaps within the chamber to constrain the insect within the field of view. The chambers were oriented such that the long axis of the body lay perpendicular to the beam path, providing either lateral or dorsoventral views.

CO_2 _emission was monitored by a flow-through respirometry system from Sable Systems International (SSI, Las Vegas, NV, USA). Room air was scrubbed of CO_2 _and H_2_O using a Drierite/Ascarite/Drierite column and pushed through the system using a pump (TR-SS3, SSI). Flow rate (100 ml/min for all species except *D. virilis*, 50 ml/min) was maintained via a mass flow controller (SSI MFC-2 using a Sierra Instruments mass flow control valve). CO_2 _exiting the insect chamber was measured by a gas analyzer (LI-7000, Li-Cor, Lincoln, NE, USA). Chamber washout times were on the order of 6–12 s. CO_2 _data were output to a computer via UI-2 (SSI) and recorded using ExpeData software (SSI). Pre-beam CO_2 _emission was typically recorded for 5–10 minutes before opening the x-ray shutter. For survivorship trials, insects were exposed to x-rays on the head, thorax, or abdomen until they clearly showed a respiratory signature that we infer to be respiratory function damage; otherwise, trials were ended after 2 hrs. CO_2 _emission was also monitored post-beam for up to 30 minutes. For behavioral trials, the insects were exposed for a fixed amount of time, then removed from the chamber and tested for the presence/absence of righting behavior, defensive behavior, and locomotor ability. All trials were conducted at room temperature (21–22°C). Data were analyzed using ExpeData and LabAnalyst X (Mark Chappell, University of California Riverside, CA, USA) software packages.

### Feeding

To demonstrate the use of x-rays to visualize internal food movement during ingestion and digestion, beetles (*Platynus decentis*) were fed macerated insects mixed with fine particles of CdWO_4_, and butterflies (*Pieris rapae*) were fed sugar solutions laced with an iodine contrast agent (Isovue, Bracco Diagnostics, NJ, USA). Animals were held in place by securing the body to a microscope cover slip (beetles) or by a mounted clamp attached to the wings (butterflies). These examples illustrate the use of contrast agents to visualize internal food transport. The fine particles of CdWO_4 _had a significantly higher absorption over the entire x-ray energy range used in this study and appeared darker than the surrounding soft tissue. In the case of the iodine solution, differences in x-ray absorption at the nominal setting (25 keV) were minimal. To maximize contrast between the iodine and the surrounding anatomy, we used an energy just above the K-absorption edge (33.2 keV for iodine), where absorption increased dramatically. Because in general the use of higher energy x-rays results in an overall lower contrast for soft tissue (Figure 3), this technique is most applicable for cases where it is more important to track internal movements of food than to visualize clearly the surrounding insect anatomy. The use of simultaneous x-ray images above and below the K-edge to improve visualization of the contrast agent is possible, but would require a more complicated set of x-ray optics. Furthermore, it would imply a doubling of the x-ray dose to the animal.

For contrast agents, iodine is more suitable for fluids whereas CdWO_4 _is more suitable for solids. Although we have not investigated the toxicity of iodine versus CdWO_4 _in insects, we speculate that iodine is less harmful because Isovue is used for human medical diagnosis, and it is well known that cadmium is toxic [35]. We chose a cadmium compound for its convenience, but other high electron density materials in powder form (such as silica or lead) can be used to provide radio-opacity with lower toxicity.

### Temperature

Possible change in temperature in the insect due to the absorption of x-rays was measured separately with two methods. First, an implanted 0.01 mm copper-constantan thermocouple and thermocouple thermometer (0.1 K resolution, Physitemp Instruments, Inc., NY, USA) were used to measure internal abdominal temperature in an adult grasshopper (*Schistocerca gregaria*) while it was irradiated for up to 10 minutes on the thorax. To test if local heating occurred at the site of irradiation, an infrared camera (Inframetrics 760, 0.1 K resolution, American Infrared, NY, USA) was used to visualize temperature change in the head of three beetles (*Platynus decentis*) and one grasshopper (*Schistocerca gregaria*).

### Data analysis

We used two metrics to quantify the effect of the x-rays on the CO_2 _emission patterns. To assess immediate effects of the beam, we compared CO_2 _emission rates in the 2 minutes before 'beam on' to those 2 minutes after 'beam on' (Equation 2). We defined the ratio (*R*_2 *min*_) as:

R2min⁡=〈Ebeam−on〉2min⁡〈Epre−beam〉2min⁡     (2)
 MathType@MTEF@5@5@+=feaafiart1ev1aaatCvAUfKttLearuWrP9MDH5MBPbIqV92AaeXatLxBI9gBaebbnrfifHhDYfgasaacH8akY=wiFfYdH8Gipec8Eeeu0xXdbba9frFj0=OqFfea0dXdd9vqai=hGuQ8kuc9pgc9s8qqaq=dirpe0xb9q8qiLsFr0=vr0=vr0dc8meaabaqaciaacaGaaeqabaqabeGadaaakeaacqWGsbGudaWgaaWcbaGaeGOmaiJagiyBa0MaeiyAaKMaeiOBa4gabeaakiabg2da9maalaaabaWaaaWabeaacqWGfbqrdaWgaaWcbaGaemOyaiMaemyzauMaemyyaeMaemyBa0MaeyOeI0Iaem4Ba8MaemOBa4gabeaaaOGaayzkJiaawQYiamaaBaaaleaacqaIYaGmcyGGTbqBcqGGPbqAcqGGUbGBaeqaaaGcbaWaaaWabeaacqWGfbqrdaWgaaWcbaGaemiCaaNaemOCaiNaemyzauMaeyOeI0IaemOyaiMaemyzauMaemyyaeMaemyBa0gabeaaaOGaayzkJiaawQYiamaaBaaaleaacqaIYaGmcyGGTbqBcqGGPbqAcqGGUbGBaeqaaaaakiaaxMaacaWLjaWaaeWaaeaacqaIYaGmaiaawIcacaGLPaaaaaa@5C2B@

where 〈*E*_*pre-beam*_〉_2 min _and 〈*E*_*beam-on*_〉_2 min _are, respectively, the CO_2 _emission rates (μl/hr) averaged over the 2 minutes immediately prior and after the x-rays (25 keV) were turned on. Second, to assess the duration required for damage to occur due to x-rays, we identified a major change in the CO_2 _emission pattern within each species, which we refer to as the respiratory signature (RS; Figure 6). The RS was chosen for its repeatability; by the time of the RS, major (and likely irreversible) damage has occurred. This time interval, between when the beam first hit the insect and the RS, is the time to respiratory signature (TTRS).

Image quality and the corresponding photon fluxes and average power densities for 25 keV x-rays used in this study are shown in Figure 4. The incident photon fluxes were chosen for an approximate factor of two change in intensity between each setting. At 25 keV, a 1-mm thick water sample (over the entire beam area) would absorb about 3% of the incident beam energy [36]. Absorbed power for a volume of insect that is irradiated can thus be estimated by Equation 3:

Absorbed power = 0.03 × *D *× *V *    (3)

where *D *is the incident beam power density (μW/mm^2^) and *V *is the irradiated volume in mm^3^. Assuming an incident power density of 80 μW/mm^2 ^and an ant head of volume 3 mm^3^, the estimated absorbed power is 7.2 μW and the absorbed dose rate (absorbed power per unit mass) is 2.4 Gy/s. X-ray absorbed powers were measured for each species (Table 2); these values are consistent with the theoretical estimates.

**Table 2:** Effect of duration of x-ray exposure to head on motor control abilities. m/n denotes m animals behaving normally out of n animal trials. Asterisks denote partially limited response (e.g., slow response).

		**Exposure time (min) with 25 keV x-rays, 80 μW/mm**^2^									
		
**Species**	**Behavior**	**2**	**4**	**5**	**6**	**8**	**10**	**12**	**15**	**25**	**120+**
Ant	Righting	2/2	2/2		1/2	1*/2	2*/2	1*/2			
	Defense	2/2	2/2		1/2	1/2	1*/2	1*/2			
	Walking	2/2	2/2		2*/2	1*/2	0/2	0/2			

Fruit fly	Righting	1/1	2/2		2/2	2/2	0/2	1*/2			
	Walking	1/1	2/2		2*/2	2*/2	0/2	1*/2			
	Flight	1/1	2/2		2/2	2/2	0/2	1*/2			

Beetle	Righting			1/1			1/1		0/1	0/1	
	Walking			1/1			1*/1		1*/1	0/1	
	Running			1/1			1*/1		0/1	0/1	

Grasshopper	Righting										2/2
	Hopping										2/2
	Flight										2/2

Metabolic rate calculations were based on averages of all available prebeam CO_2 _recordings for each of the four species. Conversion from CO_2 _output to metabolic rate assumed respiratory quotients (RQ) of 1.0, 0.8, 0.7, and 1.0 and energy equivalences of 20.1, 24.5, 27.6 and 21.2 J/ml of CO_2 _for grasshoppers [37], beetles [38], ants [39], and fruit flies [40], respectively.

## Abbreviations

DGC, discontinuous gas exchange  

D, incident beam power density  

d, sample-detector distance  

E, x-ray energy  

I_inc_, incident beam intensity  

L, source-sample distance  

λ, x-ray wavelength  

ρ, sample density  

RQ, respiratory quotient  

RS, respiratory signal  

R(x,y), effective detector resolution  

σ_s,_ source size  

TTRS, time to respiratory signal  

V, volume  

x-y, image plane  

z, beam direction  

## Competing interests

The authors declare that they have no competing interests.

## Authors' contributions

The scope of this paper was conceived by all authors. JS and JW collected most of the data. JS and WKL performed the analyses. JS and WKL drafted the manuscript, and JH and MW contributed to the writing. All authors read and approved the final manuscript.

## Supplementary Material

Additional File 1 **Rhythmic compressive movements in the tracheal system in the carabid beetle *Platynus decentis*, demonstrating the utility of phase-contrast synchrotron imaging for studies of respiratory dynamics in small animals**. View (1.3 × 1.0 mm) is a dorsoventral projection through prothorax of a beetle (mass ~ 45 mg) using monochromatic x-rays (25 keV). The midline of the beetle lies on the right side of the video between the two coxae (large circular structures, bottom right). Collapse and reinflation of the air-filled tracheal tubes can be seen in the majority of the tubes in view. The smallest tracheal tubes that can be seen are about 10 μm in diameter; tracheoles (<1 μm diameter) are too small to be resolved. The circle and dark opaque spots the upper right are an air bubble and particles in the esophagus, respectively; note that they move anteriorly and posteriorly during the compression of the tracheal tubes. The white and dark spots that do not move with the beetle movement are artifacts due to the incident beam and detector system. Click here for file

Additional file 2 **Passage of food bolus through the esophagus of the butterfly *Pieris rapae***. View (1.3 × 1.0 mm) is a lateral projection through the thorax of the butterfly (mass ~50 mg), with food moving from anterior (upper right) to posterior (lower left). The butterfly was feeding on a mixture of sugar water and iodine compound (Isovue). X-ray energy (33.2 keV) was tuned just above the K-edge for iodine, making the food bolus appear dark. This clip demonstrates how synchrotron imaging can be used to visualize internal food transport during feeding in small animals. Note that the esophagus is collapsed until the bolus passes through; the light structure running along the same diagonal axis is a tracheal tube. From this clip, it can be seen that the bolus is tapered at both ends and is transported at a speed of ~1.5 mm/s. Click here for file

Additional file 3 **Movements of the foregut and gut contents of the carabid beetle *Pterostichus stygicus***. View (3.3 × 2.5 mm) is a dorsoventral projection through the pterothorax, posterior to the mesocoxae (circular structures seen at top of image). The beetle (mass ~210 mg) was fed macerated larva sprinkled with cadmium powder to increase x-ray (25 keV) absorption contrast; the gut boundaries and food movement can only be seen in places with cadmium powder. In this sequence, the crop (bag-like structure, center left) is squeezed anteriorly and then slowly settles back into its initial orientation. Mixing movements and peristalsis of the proventriculus (cylindrical structure, right side) can also be seen. Note that the proventriculus is closed, preventing food from moving posteriorly into the midgut. Dark bands on the left side of the video are artifacts from the incident beam. Click here for file
